# Circulating Tumor DNA Markers for Early Progression on Fulvestrant With or Without Palbociclib in ER+ Advanced Breast Cancer

**DOI:** 10.1093/jnci/djaa087

**Published:** 2020-06-17

**Authors:** Ben O’Leary, Rosalind J Cutts, Xin Huang, Sarah Hrebien, Yuan Liu, Fabrice André, Sibylle Loibl, Sherene Loi, Isaac Garcia-Murillas, Massimo Cristofanilli, Cynthia Huang Bartlett, Nicholas C Turner

**Affiliations:** 1 Breast Cancer Now Toby Robins Research Centre, The Institute of Cancer Research, London, UK; 2 Breast Unit, Royal Marsden Hospital, London, UK; 3 Pfizer, New York, NY, USA; 4 Department of Medical Oncology, Institut Gustave Roussy, Université Paris Sud, Villejuif, France; 5 German Breast Group, Martin Behaim-Strasse 12, Neu-Isenburg, Germany; 6 Division of Research and Cancer Medicine, Peter MacCallum Cancer Centre, University of Melbourne, Melbourne, Australia; 7 Robert H Lurie Comprehensive Cancer Centre, Feinberg School of Medicine, Chicago, IL, USA

## Abstract

**Background:**

There are no established molecular biomarkers for patients with breast cancer receiving combination endocrine and CDK4/6 inhibitor (CDK4/6i). We aimed to determine whether genomic markers in circulating tumor DNA (ctDNA) can identify patients at higher risk of early progression on fulvestrant therapy with or without palbociclib, a CDK4/6i.

**Methods:**

PALOMA-3 was a phase III, multicenter, double-blind randomized controlled trial of palbociclib plus fulvestrant (n = 347) vs placebo plus fulvestrant (n = 174) in patients with endocrine-pretreated estrogen receptor–positive (ER+) breast cancer. Pretreatment plasma samples from 459 patients were analyzed for mutations in 17 genes, copy number in 14 genes, and circulating tumor fraction. Progression-free survival (PFS) was compared in patients with circulating tumor fraction above or below a prespecified cutoff of 10% and with or without a specific genomic alteration. All statistical tests were 2-sided.

**Results:**

Patients with high ctDNA fraction had worse PFS on both palbociclib plus fulvestrant (hazard ratio [HR] = 1.62, 95% confidence interval [CI] = 1.17 to 2.24; *P* = .004) and placebo plus fulvestrant (HR = 1.77, 95% CI = 1.21 to 2.59; *P* = .004). In multivariable analysis, high-circulating tumor fraction was associated with worse PFS (HR = 1.20 per 10% increase in tumor fraction, 95% CI = 1.09 to 1.32; *P* < .001), as was *TP53* mutation (HR = 1.84, 95% CI = 1.27 to 2.65; *P* = .001) and *FGFR1* amplification (HR = 2.91, 95% CI = 1.61 to 5.25; *P* < .001). No interaction with treatment randomization was observed.

**Conclusions:**

Pretreatment ctDNA identified a group of high-risk patients with poor clinical outcome despite the addition of CDK4/6 inhibition. These patients might benefit from inclusion in future trials of escalating treatment, with therapies that may be active in these genomic contexts.

CDK4/6 inhibitors (CDK4/6i) now play a key role in the treatment of advanced, estrogen receptor–positive (ER+) breast cancers (1), with established efficacy in combination with endocrine therapy in both first- and second-line treatment (2–8). However, a substantial proportion of patients progress early on treatment, and there is a clinical need to identify patients at risk of early progression.

There are a number of established molecular markers associated with poor outcome in early ER+ breast cancer, most notably the risk classifiers based on gene expression assessed in tumor biopsies, which are now routinely used to augment clinical decision making (9). Genomic markers other than *HER2* amplification associated with poorer outcome in primary disease include mutations in *TP53* (10,11), amplifications in *FGFR1* (12), which may contribute to endocrine therapy resistance (13), and amplification of *MYC* (14). Less is known of the associations between common genomic aberrations in advanced ER+ breast cancer and clinical outcome, particularly in the updated therapeutic landscape that includes combination CDK4/6i treatments.

Recent work has identified a number of potential genomic mechanisms of resistance to CDK4/6i, notably amplification of *CCNE1*, mutations in *FAT1*, CDK6 overexpression, and loss of *RB1* (15,16), with emerging data for immune signatures and other oncogenic signaling (17,18). Of these, clinical data support acquisition of *RB1* mutations in a minority of cancers progressing on CDK4/6i (19,20), with preexisting loss of functional RB1 associated with poor prognosis on CDK4/6i therapy. Loss of *FAT1* was also associated with poor outcome on CDK4/6i therapy (21), although inactivating mutations in *FAT1* are rare in advanced ER+ breast cancer. We have shown previously that mutations in *PIK3CA* and *ESR1* in advanced ER+ breast cancer previously treated with endocrine therapy do not predict response to palbociclib (22).

Circulating tumor DNA (ctDNA) is found in the plasma of a substantial majority of patients with advanced cancer and presents a source of cancer DNA for noninvasive analysis of tumor somatic genetic features. In addition, circulating tumor fraction, the fraction of plasma DNA that is derived from the tumor, may be a biological marker that reports on both tumor bulk and tumor aggressiveness (23) and is associated with poorer clinical outcome in triple-negative breast cancer (24).

In conducting this analysis, we hypothesized that genomic aberrations identified at baseline, including mutations, copy number, and circulating tumor fraction, could be predictive or prognostic of clinical outcome for patients with advanced ER+ breast cancer receiving fulvestrant with or without palbociclib. We investigated this using a multimodal ctDNA sequencing analysis of plasma DNA from the PALOMA-3 trial.

## Methods

Full details of the methods can be found in the Supplementary Methods (available online).

### Study Design and Patients

The design of the PALOMA-3 trial (NCT01942135) and clinical outcome data has been previously reported (2). Patients with advanced ER+ breast cancer that had previously progressed on endocrine therapy were randomized 2:1 to receive palbociclib plus fulvestrant or placebo plus fulvestrant.

### Plasma Collection and DNA Extraction

Blood was collected in EDTA tubes on day 1 of treatment and, within 30 minutes, was centrifuged at 3000 g for 10 minutes before plasma separation. Samples were then stored at -80°C prior to DNA extraction. DNA concentration was estimated using a droplet digital polymerase chain reaction (PCR) assay directed at *RPPH1* on the BioRad QX200.

### Sequencing and Digital PCR

Mutations were assessed in baseline plasma DNA using a previously reported targeted error-corrected sequencing approach, utilizing a bespoke bioinformatic pipeline incorporating integrated digital error suppression (19,25). The targeted panel included 17 genes, with all coding exons of *CDK4*, *CDK6*, *CDKN1A*, *CDKN1B*, *RB1*, and *NF1*; exons 5-8 of *TP53*, and mutation hotspots in *AKT1*, *ERBB2*, *ESR1*, *PIK3CA*, *FGFR1*, *FGFR2*, *FGFR3*, *KRAS*, *HRAS*, and *NRAS*. Of the baseline plasma DNA sequencing, 195 patients were previously sequenced to compare mutational profile with end-of-treatment progression plasma (19), with an additional previously unreported 136 patients’ baseline plasma DNA sequenced for the comprehensive baseline analysis presented here. Digital PCR had been previously performed on the baseline plasma DNA samples for *PIK3CA* and *ESR1* mutation (26).

Circulating tumor fraction was assessed using a previously reported bespoke targeted amplicon panel including prevalent heterozygous single-nucleotide polymorphisms in 8 regions commonly lost in breast cancer, additionally with amplicons assessing for loss or loss of heterozygosity of *RB1*, *PTEN*, and *CDKN2A* and for gain of *ERBB2*, *EGFR*, *PIK3CA*, *ESR1*, *CDK4*, *FGFR1*, *FGFR2*, *MYC*, *MCL1*, *CCND1*, and *CCNE1* (19). Comparison with tumor fraction estimated from low-pass whole-genome sequencing was performed in 19 samples sequenced with tumor fraction estimated using ichorCNA (23).

### Statistical Analysis

The primary outcome of this study was to identify potential prognostic and predictive factors for progression-free survival (PFS) within both treatment arms. PALOMA-3 was designed and powered for a clinical endpoint and, as such, was not specifically powered for a translational analysis. Survival analyses to associate PFS with genomic aberrations were performed with Cox proportional hazards models, with calculation of hazard ratios (HR), 95% confidence intervals (CI), and log-rank *P* values. Proportionality was assessed using the method described by Grambsch and Thernau (27). For circulating tumor fraction analysis, a 10% cutoff was prespecified for association with PFS as previously used in the literature (23,24). To explore the potential statistical significance of genomic alterations, an initial univariable analysis in each treatment arm was planned to be followed by a multivariable analysis incorporating treatment as a variable to test for interaction. Associations of clinical and pathological characteristics with genomic aberrations were tested with χ^2^ tests or Cochran-Armitage tests for trend. *P* values were considered statistically significant for values less than .05. The Benjamini-Hochberg approach was used to adjust for multiple comparisons. All statistical tests were 2-sided.

## Results

### Circulating Tumor Fraction and Progression-Free Survival

**Figure 1.**
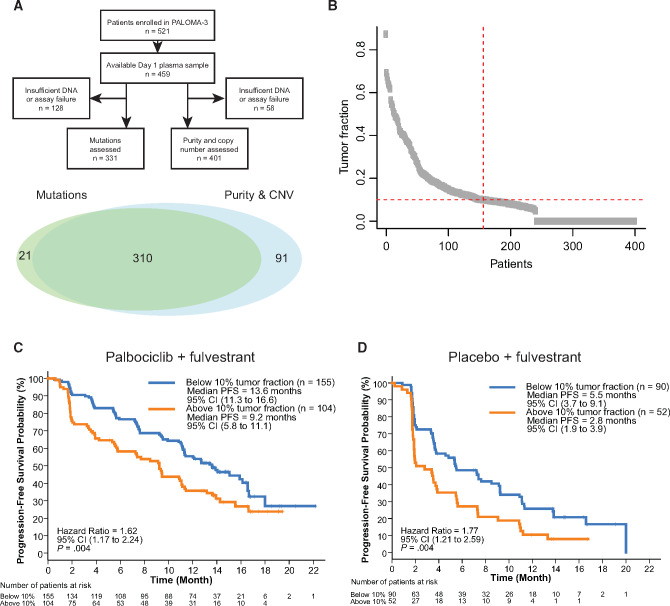
Circulating tumor fraction and progression free survival (PFS) in PALOMA-3. **A**) CONSORT and Venn diagram showing analysis of plasma samples from the PALOMA-3 trial. **B**) Distribution of detected circulating tumor fraction at baseline (n = 401). **C**) PFS for the palbociclib plus fulvestrant group (n = 259) split by circulating tumor fraction above or below 10%. **D**) PFS for the placebo plus fulvestrant group (n = 142) split by circulating tumor fraction above or below 10%. *P* values are log-rank. CI = confidence interval; CNV = copy number variant.

Of the enrolled patients with available plasma, 401 of 459 (87.4%) patients had sufficient material and subsequent library quality for circulating tumor fraction and copy number analysis, a group with outcomes representative of the overall trial population (Figure 1A;Supplementary Figure 1, available online). Circulating tumor fraction assessment was found to agree well with orthogonal assessment in a sample (n = 19) of plasma assessed for tumor fraction using low-depth, whole-genome sequencing (Pearson *r* = 0.86; *P* < .001; Supplementary Figure 2, available online) and tumor fraction correlated with *PIK3CA* allele fraction (Pearson *r* = 0.71; *P* < .001; Supplementary Figure 3, available online) and *TP53* allele fraction (Pearson *r* = 0.79; *P* < .001; Supplementary Figure 4, available online).

**Figure 2.**
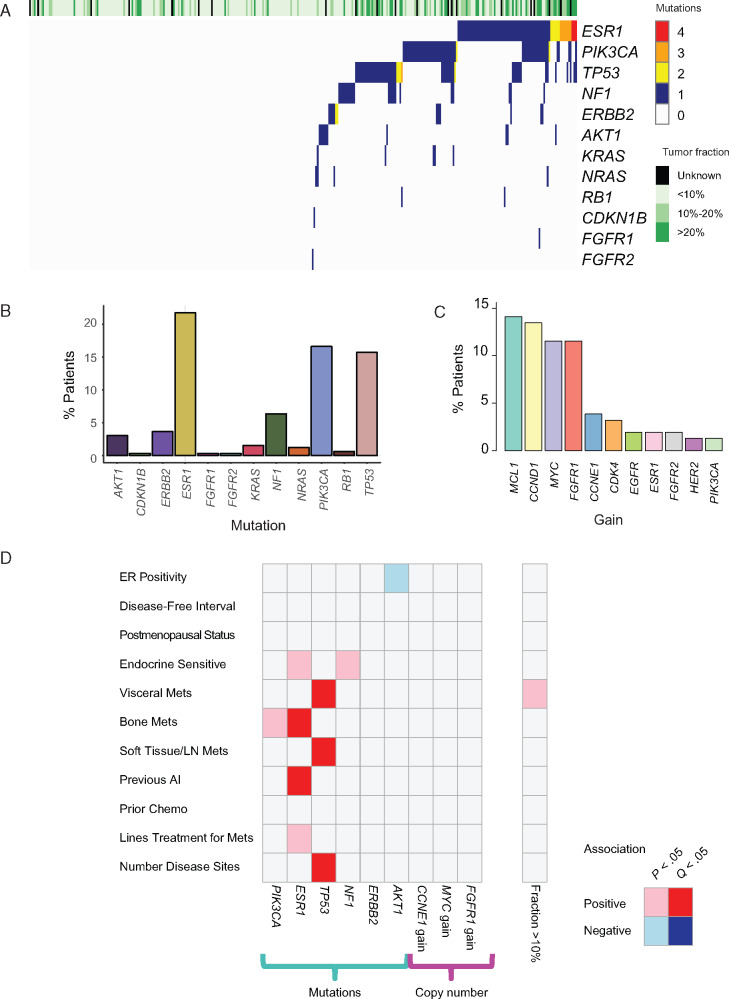
Genomic landscape of endocrine-resistant breast cancer in circulating tumor DNA analysis. **A**) Distribution and number of mutations by patient at baseline. **B**) Prevalence of mutated genes observed in the baseline plasma samples (n = 331). **C**) Prevalence of copy number gain from the subset of patients with greater than 10% circulating tumor fraction. **D**) Associations between clinic-pathological characteristics and ctDNA genomic features. *P* values calculated with χ^2^ if categorical or Cochran-Armitage tests if ordinal, corrected using Benjamini-Hochberg method. CNV = copy number variant; ctDNA = circulating tumor DNA; ER = estrogen receptor; LN = lymph node.

A high-circulating tumor fraction (>10% fraction, prespecified) was observed in 38.9% (156 of 401) patients (Figure 1B). In the palbociclib plus fulvestrant group, median PFS in patients with circulating tumor fraction of more than 10% was 9.2 months (95% CI = 5.8 to 11.1) and for those with circulating tumor fraction of no more than 10% was 13.6 months (95% CI =11.3 to 16.6; HR = 1.62, 95% CI = 1.17 to 2.24; log-rank *P* = .004; Figure 1C). In the placebo plus fulvestrant group, median PFS in patients with circulating tumor fraction of more than 10% was 2.8 months (95% CI = 1.9 to 3.9) and with circulating tumor fraction of no more than 10% was 5.5 months (95% CI = 3.7 to 9.1, HR = 1.77, 95% CI = 1.21 to 2.59; log-rank *P* = .004; Figure 1D). In an exploratory analysis using discrete cutoffs, circulating tumor fractions of more than 20% were associated with increasingly worse PFS (Supplementary Figure 5, available online).

### Genomic Analysis in Baseline ctDNA and Association With Clinical Characteristics

Of the 521 patients enrolled in the study, 331 of 521 (63.5%) had sufficient material and subsequent library quality for mutation analysis by sequencing, with this population also representative of the overall trial (Figure 1A;Supplementary Figure 6, available online). The most commonly mutated gene was *ESR1* (72 of 331 [21.8%]; Figures 2, A and B), with a comparable prevalence of *PIK3CA* mutation (55 of 331 [16.6%]) and *TP53* (52 of 331 [15.7%]). Smaller proportions of patients had mutations in *NF1* (21 of 331 [6.3%]), *ERBB2* (12 of 331 [3.6%]), and *AKT1* (10 of 331 [3.0%]). Mutations in *ESR1* were polyclonal in a subset of patients (16 of 72 [22.2%]; Figure 2A).

Detection of copy number aberrations (CNAs) is technically challenging in samples with low circulating tumor fraction, and we assessed the prevalence of CNAs only in the group with more than 10% circulating tumor fraction. The most frequently observed gains from the genes included in the panel were *MCL1*, *CCND1*, *MYC*, and *FGFR1* (Figure 2C), and there was evidence of copy number loss and/or loss of heterozygosity in similar proportions of *RB1* (27 of 156 [17.3%]), *PTEN* (30 of 156 [19.2%]), and *CDKN2A* (34 of 156 [22.0%]).

Having established the landscape of genomic aberrations in ctDNA at baseline, we assessed associations with clinical characteristics (Figure 2D). A positive association was observed between *ESR1* mutation and previous endocrine sensitivity (χ^2^*P* = .015), previous aromatase inhibitor exposure (χ^2^*P* = .002), bone metastases (χ^2^*P* = .005), and a number of all previous lines of treatment for metastatic disease (Cochran-Armitage *P* = .02), associations similar to those previously reported using digital PCR analysis (Supplementary Figure 7, available online) (22). Prior aromatase inhibitor therapy (χ^2^*Q* = .021) and bone metastases (χ^2^*Q* = .028) remained statistically significant after correction for multiple testing using the Benjamini-Hochberg method. *TP53* mutations were positively associated with visceral metastases (χ^2^*Q* = .046), soft tissue and lymph node metastases (χ^2^*Q* = .042), and a number of disease sites (Cochran-Armitage *Q* = .009). No other mutations or copy number changes were statistically significantly associated with a particular clinical characteristic after correction for multiple testing. There was no detectable association between circulating tumor fraction of more than 10% and clinical and pathological features, after correcting for multiple comparisons (Figure 2D). Mutations in specific genes associated with higher circulating tumor fractions in patients—this was statistically significant for the most prevalent mutations, in *PIK3CA*, *TP53*, and *ESR1*, most likely simply demonstrating that higher circulating tumor fraction means mutation detection in ctDNA is more likely (Supplementary Figure 8, available online).

### Genomic Analysis in Baseline ctDNA and Progression-Free Survival

**Figure 3.**
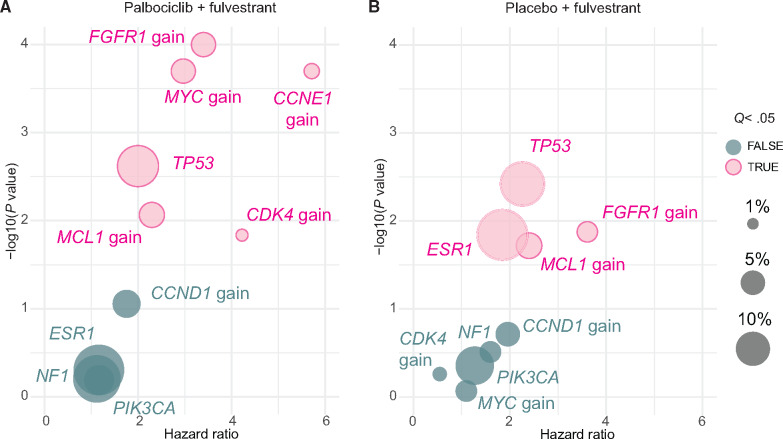
Association between ctDNA genomic features and progression-free survival (PFS). **A)** Univariable analyses of PFS by detected genomic aberrations for the palbociclib plus fulvestrant group. **B**) Univariable analyses of PFS by detected genomic aberrations for the placebo plus fulvestrant group. The size of bubble indicates the prevalence within the treatment group. *P* values are log-rank. *P* value correction is with the Benjamini-Hochberg method to give *Q* values. ctDNA = circulating tumor DNA.

We next analyzed associations between mutations and copy number changes and PFS, initially with both treatment groups separately in a univariable analysis. In the group of patients treated with palbociclib plus fulvestrant (n = 223 for mutations; n = 259 for copy number; Figure 3A), *TP53* mutations were associated with worse PFS (HR = 2.00, 95% CI = 1.28 to 3.12; log-rank *P* = .002). Multiple copy number gains were associated with poorer PFS including *MCL1* gain (HR = 2.29, 95% CI = 1.24 to 4.26; log-rank *Q* = .014), *FGFR1* gain (HR = 3.40, 95% CI = 1.91 to 6.04; log-rank *Q* < .001), *MYC* gain (HR = 2.97, 95% CI = 1.67 to 5.26; log-rank *Q* < .001), *CDK4* gain (HR = 4.22, 95% CI = 1.33 to 13.41; log-rank *Q* = .021), and *CCNE1* gain (HR = 5.71, 95% CI = 2.30 to 14.21; log-rank *Q* < .001). Loss and/or loss of heterozygosity of *RB1*, *PTEN*, and *CDKN2A* were also associated with worse prognosis (Supplementary Figures 9 and 10, available online).

In the group of patients treated with placebo plus fulvestrant (n = 108 for mutations; 142 for copy number; Figure 3B), *TP53* mutations (HR = 2.26, 95% CI = 1.30 to 3.93; log-rank *Q* = .026) and *ESR1* mutations (HR = 1.85, 95% CI = 1.13 to 3.02; log-rank *Q* = .047) were associated with worse PFS after correction for multiple testing using the Benjamini-Hochberg method. Copy number gain (amplification) of *FGFR1* (HR = 3.61, 95% CI = 1.31 to 9.97; log-rank *Q* = .047) and *MCL1* (HR = 2.40, 95% CI = 1.15 to 4.99; log-rank *Q* = .05) were associated with worse PFS. With a 2:1 randomization, the placebo plus fulvestrant group was relatively small, making direct comparisons between treatment groups challenging, and there were no individual aberrations that had a statistically significant interaction *P* value with treatment.

**Figure 4.**
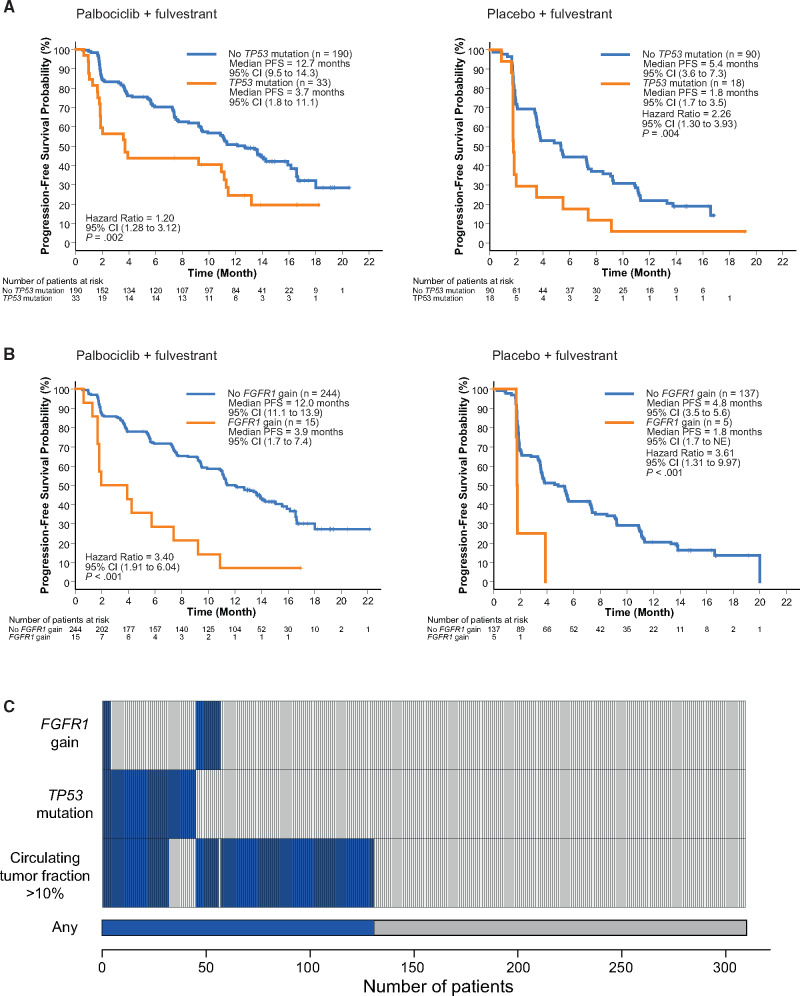
*TP53* mutation, *FGFR1* amplification, and progression-free survival (PFS). **A**) PFS by detected *TP53* mutation for the palbociclib plus fulvestrant and placebo plus fulvestrant groups. **B)** PFS by detected *FGFR1* gain for the palbociclib plus fulvestrant and placebo plus fulvestrant arms. **C**) Per patient distribution of detected *TP53* mutation, *FGFR1* gain, and circulating tumor fraction from the n = 310 patients with all of mutations, copy number, and circulating tumor fraction data available. Hazard ratio univariable analysis; P values are log-rank. CI = confidence interval; NE = not estimable.

As increased circulating tumor fraction was required to detect copy number changes in plasma, and higher circulating tumor fraction was associated with worse PFS, we performed a multivariable survival analysis (see Methods and Table 1). Circulating tumor fraction remained statistically significant in the model (HR = 1.20 per 10% increase in tumor fraction, 95% CI = 1.09 to 1.32; log-rank *P* < .001), along with *TP53* mutation (HR = 1.84, 95% CI = 1.27 to 2.65; log-rank *P* = .001) and *FGFR1* gain (HR = 2.91, 95% CI = 1.61 to 5.25; log-rank *P* < .001). Patients with *TP53* mutations and *FGFR1* amplifications had a very poor median PFS on palbociclib and fulvestrant of 3.7 months and 3.9 months, respectively (Figure 4, A and B). There was no statistically significant interaction for any genomic aberration with treatment randomization. For the analyzed cohort, ctDNA analysis identified at least 1 of the 3 poor prognosis factors, circulating tumor fraction greater than  10%, *TP53* mutation, or *FGFR1* gain in 42.3% (131 of 310) patients (Figure 4C).

**Table 1.** Multivariable analysis of the association between circulating tumor DNA genomic features and progression free survival^a^

Variable	Hazard ratio (95% CI)	*P* ^b^
*FGFR1* gain	2.91 (1.61 to 5.25)	<.001
*TP53* mutation	1.84 (1.27 to 2.65)	.001
ctDNA tumor fraction	1.20 (1.09 to 1.32)	<.001
Palbociclib	0.43 (0.32 to 0.57)	<.001

^a^ Treatment and circulating tumor fraction are included as variables in the model, the latter as a continuous variable calculated per unit 10% increase. *P* values are log-rank. The table includes only those factors remaining statistically significant with a *P* < .05. The model was constructed using forward stepwise selection including all genomic alterations that were statistically significant with log-rank *P* < .05 in both of the treatment arms (Figure 3), specifically gain of *FGFR1*, *CCNE1*, *MCL1*, *MYC*, *CDK4*; loss of *RB1*, *CDKN2A*, *PTEN*; and mutation in *TP53* and *ESR1*. CI = confidence interval; ctDNA = circulating tumor DNA.

^b^ Two-sided log-rank.

## Discussion

Combinations of CDK4/6i and endocrine therapy are standard of care in advanced ER+ breast cancer. There are few molecular markers available to identify patients at risk of early progression, where increased monitoring to detect progression may be appropriate and for whom research efforts might be focused to improve outcomes. We have previously published work from the PALOMA-3 trial examining the evolution of resistance (19). Here, we build on this by using a multimodal ctDNA sequencing analysis of all of the baseline plasma samples to assess for predictive and prognostic genomic features, greatly expanding the range of baseline genomic alterations from our previous work on *ESR1* and *PIK3CA* using digital PCR (22). We did not identify any predictive genomic alterations, but circulating tumor fraction, *TP53* mutation, and *FGFR1* gain were each independently associated with risk of early relapse for both fulvestrant alone and fulvestrant plus palbociclib treatments. Approximately half of the patients with *TP53* mutation or *FGFR1* gain detected in plasma DNA had progressed by 2 months, despite the addition of a CDK4/6i. Combined, these genomic markers identify a subset of the patients (42.3%), a group who may benefit from augmented treatment strategies.

Broadly, there was strong agreement between the estimated circulating tumor fraction and those mutations expected to be commonly clonal, such as in *PIK3CA* and *TP53*, although the association was weaker at lower mutation allele fractions, likely reflecting subclonal mutations and stochastic effects (Supplementary Figures 2, 3, and 4, available online). Circulating tumor fraction was strongly associated with adverse PFS in both treatment groups in the PALOMA-3 study (Figure 1) and emerged as an independent prognostic factor in the multivariable analysis—the first demonstration of this association in ER+ breast cancer. Although levels of ctDNA are associated with stage and tumor burden (28), they are not simply a surrogate for tumor volume and are associated with proliferation (26,29–32), and it is likely that circulating tumor fraction is an independent prognostic marker because of the collective effect of all these elements. Consistent with prior reports, we found no association of ctDNA fraction with the number of disease sites. Our findings are also consistent with observations in triple-negative breast cancer (24), suggesting such analysis could become a general tool in stratifying risk for breast cancer patients. In addition, given that circulating tumor fraction is associated with the ability to detect genomic aberrations in ctDNA analysis, our analysis highlights the importance of considering circulating tumor fraction when validating associations between ctDNA detected mutations or copy number changes and clinical outcomes.

We did not identify any genomic alterations that were predictive for outcome on palbociclib. In the univariable analysis, some alterations were observed to have a consistent association with PFS in both arms, notably *TP53* and *FGFR1* gain (Figure 3), with others appearing in only one, such as *CCNE1* and *CDK4* gain in the palbociclib arm and *ESR1* mutation in the fulvestrant-alone arm. However, no statistically significant treatment interaction effect was observed with any alteration. Some of these alterations, notably *CCNE1* gain, which was associated with marked poor prognosis in the palbociclib plus fulvestrant group (Figure 3), remain plausible palbociclib resistance markers with prediction analysis underpowered because of low prevalence. For prognosis, only *TP53* mutation and *FGFR1* gain remained statistically significantly associated with worse outcome once treatment and circulating tumor fraction were taken into account.

*TP53* is one of the most commonly mutated genes in breast cancer (33), observed at a higher prevalence in luminal B cancers as compared with luminal A cancers (33). In this analysis, *TP53* mutations were associated with a distinct clinical phenotype characterized by more sites of metastases and more prevalent visceral and soft tissue and lymph node metastases. *TP53* mutations associate with poorer clinical outcome in ER+ primary breast cancer (10,11) and endocrine resistance (34). Our work suggests that the aggressive biology for *TP53* mutant ER+ breast cancer continues in the advanced setting, with the association between *TP53* mutation and poor outcome in both treatment arms of the PALOMA-3 trial demonstrating consistency of this finding across 2 different treatments and raising the question of considering this subset of breast cancer a distinct clinical entity.

*FGFR1* amplification emerged as independently associated with early progression. *FGFR1* amplification is associated with endocrine resistance (13), and with no observed interaction effect with treatment, this finding predominantly suggests resistance to the fulvestrant backbone element of the combination. As with *TP53* mutation, a similar effect was observed in the separate treatment arms. Nevertheless, recent data has highlighted a potential role for FGFR signaling in resistance to CDK4/6i (35). This suggests the potential of FGFR inhibitors, in particular in cancers with high-level *FGFR1* amplification (36) that would be more readily detectable in ctDNA, to enhance treatment efficacy. However, the *FGFR1* 8p11/12 amplicon is often broad, with *FGFR1* signaling likely a driver only in a subset of cancers (37).

This report has a number of important limitations. Although we were able to assess and account for circulating tumor fraction accurately above 10%, robust assessment of tumor fractions below 10% was not possible, and we are unable to ascertain the potential impact of lower cutoffs. Calling copy number is challenging in plasma DNA sequencing, and the number of tumors with copy number changes has been undercalled; amplifications are only detectable in tumors with high tumor fraction or in cancers with lower tumor fractions when high levels of copy number are present in the tumor. Genomic loss is even harder to assess in plasma DNA, restricted to cancers with the highest tumor purity. Lastly, *TP53* mutations are also found in clonal hematopoiesis (38), and without direct analysis of matching buffy coat for the plasma samples, we are unable to exclude the effect of this. Prior to application in clinical trials, independent validation of these findings will be important.

In summary, using ctDNA analysis, we identify genomic features that associate with a risk of early progression on fulvestrant and palbociclib, with at least 1 feature present in 42% of patients in PALOMA-3. Validation of these findings will be required before trials assessing clinical utility are conducted (39). If the observations here can be independently validated, then patients with these features may be suitable for clinical trials of more intensive surveillance on treatment or of trials examining escalation of therapy to assess these strategies for clinical benefit.

## Funding

This work was supported by the Medical Research Council (MR/N002121/1), Breast Cancer Now with support from the Mary-Jean Mitchell Green Foundation and Pfizer. We acknowledge National Institute for Health Research funding to the Royal Marsden and Institute of Cancer Research Biomedical Research Centre. We thank Breast Cancer Now for funding this work as part of Program Funding to the Breast Cancer Now Toby Robins Research Centre.

## Notes

**Role of the funder:** The sponsor collaborated with the senior academic authors to design the clinical study and assisted with data collection, analysis, and interpretation. All authors had responsibility for the decision to submit the manuscript for publication.

**Disclosures:** BO’L—Research funding (Inst): Pfizer. YL, XH, CHB—Ex-Employment: Pfizer, Stock ownership: Pfizer. FA—Travel, Accommodation, Expenses: Novartis, Roche, GlaxoSmithKline, AstraZeneca, Research Funding (Inst): AstraZeneca, Novartis, Pfizer, Lilly, Roche. SLoib—Consulting or Advisory Role (Inst): Pfizer, Roche, Novartis, Seattle Genetics, Honoraria: Pfizer, Roche, Research Funding (Inst): Pfizer, Roche, Celgene, Amgene, Novartis, Abbvie, AstraZeneca, Seattle Genetics, Teva, Vifor Pharma. SLoi—Consulting or Advisory Role (Inst): AstraZeneca/MedImmune, Seattle Genetics, Bristol-Myers Squibb, Pfizer, Novartis, Roche/Genentech, Merck Sharp & Dohme, Research Funding (Inst): Roche/Genentech, Pfizer, Novartis, Merck, Puma Biotechnology, Bristol-Myers Squib. MC—Consulting or Advisory Role: Dompé Farmaceutici, Newomics, Vortex Biosciences, Honoraria: Dompé Farmaceutici, Pfizer. NC—Consulting or Advisory Role: Roche, Pfizer, Novartis, AstraZeneca Research Funding: Pfizer (Inst), Roche (Inst), AstraZeneca. The other authors have no conflicts of interest to discloses.

**Author contributions:** BO’L: conceived and designed the project; collected and assembled the data; data analysis and interpretation; writing manuscript. RJC: collected and assembled the data; data analysis and interpretation; writing manuscript. XH: data analysis and interpretation; writing manuscript. SH: collected and assembled the data; data analysis and interpretation; writing manuscript. YL: conceived and designed the project; collected and assembled the data; data analysis and interpretation; writing manuscript. FA: collected and assembled the data; writing manuscript. SLoib: collected and assembled the data; data analysis and interpretation; writing manuscript. SLoi: collected and assembled the data; data analysis and interpretation; writing manuscript. IG-M: conceived and designed the project; collected and assembled the data; data analysis and interpretation; writing manuscript. MC: conceived and designed the PALOMA-3 study; collected and assembled the data; data analysis and interpretation; writing manuscript. CHB: conceived and designed the PALOMA-3 study; conceived and designed the project; collected and assembled the data; data analysis and interpretation; writing manuscript. NCT: conceived and designed the PALOMA-3 study; conceived and designed the project; collected and assembled the data; data analysis and interpretation; writing manuscript.

**Acknowledgments:** We would like to thank the patients, families, and care givers who participated in the PALOMA-3 trial and all the investigators and site personnel.

## Data Availability

The data that support the findings of this study are available from the corresponding author on reasonable request.

## Supplementary Material

djaa087_Supplementary_Data
