# Supplementary material for: Circulating Tumor DNA Markers for Early Progression on Fulvestrant With or Without Palbociclib in ER+ Advanced Breast Cancer
Source: J Natl Cancer Inst. 2020 Jun 17;113(3):309–17. doi: 10.1093/jnci/djaa087 (PMC7936069; doi:10.1093/jnci/djaa087)
Supplement: djaa087_Supplementary_Data [file djaa087_supplementary_data.pdf]

Supplementary Materials

Supplementary methods

Study design and patients

The design of the PALOMA-3 trial (NCT01942135) and clinical outcome data has been previously reported[2]. Written informed consent was obtained from all participants, and genomic analysis was approved by the research ethics committee. Patients with advanced, ER+ breast cancer that had previously progressed on endocrine therapy were randomized 2:1 to receive palbociclib plus fulvestrant or placebo plus fulvestrant. Patients could have been exposed to 1 prior chemotherapy regimen. The study was approved by local institutional review boards and patients gave written informed consent to collection of blood for the purpose of ctDNA analysis at the start of treatment.

Plasma collection and DNA extraction

Blood was collected in EDTA tubes on day 1 of treatment and within 30 minutes was centrifuged at 3000g for 10 minutes before plasma separation. Samples were then stored at -80°C prior to DNA extraction using the Circulating Nucleic Acid kit (Cat No./ID: 55114) from Qiagen (Venlo, Netherlands), following a further centrifugation at 3000g for 10 minutes to pellet any debris. DNA concentration was estimated using a VIC fluorophore droplet digital PCR (ddPCR) assay directed at *RPPH1* from LifeTech (California, USA, Cat no. 4403326) run on the BioRad QX200 platform (California, USA).

Sequencing and digital PCR

Mutations were assessed in baseline plasma DNA using a previously reported targeted error-corrected sequencing approach with a calling threshold of 0.5% for SNVs, utilizing a bespoke bioinformatic pipeline incorporating integrated digital error suppression (iDES)[19, 25]. The targeted panel included 17 genes, with all coding exons of *CDK4*, *CDK6*, *CDKN1A*, *CDKN1B*, *RB1* and *NF1*, exons 5-8 of *TP53* and mutation hotspots in *AKT1*, *ERBB2*, *ESR1*, *PIK3CA*, *FGFR1*, *FGFR2*, *FGFR3*, *KRAS*, *HRAS* and *NRAS*. The library preparation incorporated two replicate multiplex PCR reactions of two primer pools with a minimum total input of 6ng of DNA, sequenced to a mean coverage of 3,276X on an Ion Proton and 12,925X on an Illumina HiSeq 2500. Of the baseline plasma DNA sequencing, 195 patients were previously sequenced to compare mutational profile with end-of-treatment progression plasma[19], with an additional previously unreported 136 patients' baseline plasma DNA sequenced for the comprehensive baseline analysis presented here. Digital PCR had been previously performed on the baseline plasma DNA samples for *PIK3CA* (n = 457) and *ESR1* mutation (n = 445) as previously reported[26].

Circulating tumor fraction was assessed using a previously reported bespoke targeted amplicon panel including prevalent heterozygous SNPs in 8 regions commonly lost in breast cancer, 22q13.31, 1p36.13, 6q27, 3p21.31, 16q24.3, 17p12, 8p23.2 and 11q23.3, independently validated down to a tumor fraction of 10%[19]. Each region featured approximately 100 SNPs for assessment of loss of heterozygosity, with approximately 130 SNPs focused around *RB1*, *PTEN* and *CDKN2A* to assess allelic loss of these genes. Loss of heterozygosity was inferred by deviation of the observed allele fraction from 0.5 beyond a coverage-dependent threshold established by sequencing unrelated germline DNA[19]. Approximately 20 amplicons (range 16 – 23) per gene were focused in 11 genes to assess copy number gain, *ERBB2*, *EGFR*, *PIK3CA*, *ESR1*, *CDK4*, *FGFR1*, *FGFR2*, *MYC*, *MCL1*, *CCND1* and *CCNE1*. Libraries were constructed with a minimum of 1ng DNA input and sequenced to a mean coverage of 1835X on an Ion Proton. Copy number gains were called using adjusted logR values in a bespoke pipeline integrating OncoCNV and utilizing a panel

of germline material, in a previously validated and published approach[19]. Comparison with tumor fraction estimated from low pass whole genome sequencing was performed in 19 samples. Libraries were constructed using the NEB NEXT Ultra II protocol and sequenced to a mean depth of 0.83X on an Illumina HiSeq 2500, tumor fraction was estimated using ichorCNA[23].

Statistical analysis

The primary outcome of this study was to identify potential prognostic and predictive factors for progression free survival within both treatment arms. PALOMA-3 was designed and powered for a clinical endpoint, to assess whether addition of palbociclib would increase progression free survival, and as such was not specifically powered for a translational analysis. Survival analyses to associate progression-free survival (PFS) with genomic aberrations were performed with Cox proportional hazards models, with calculation of hazard ratios, 95% confidence intervals and logrank p values. For circulating tumor fraction analysis a 10% cut-off was pre-specified for association with PFS as previously used in the literature [23, 24]. To explore the potential significance of genomic alterations an initial univariable analysis in each treatment arm was planned, to be followed by a multivariable analysis incorporating treatment as a variable to test for interaction. Univariable association of individual genomic aberrations with PFS by treatment arm was limited to those aberrations where at least 6 patients were identified with the aberration. A subsequent multi-variable analysis was conducted to adjust for associations between different genomic aberrations and circulating tumor fraction with PFS in the 310 patients in whom both mutation and copy number assessment were performed. The multi-variable analysis was conducted on both treatment groups combined, with the genomic aberrations that were significant in a univariable analysis, including circulating tumor fraction as a continuous variable with unit increases of 10% and treatment as variables, with an interaction term

added to assess association between aberrations and palbociclib treatment effect. An exploratory multi-variable analysis was also conducted with clinical and pathological features. Associations of clinical and pathological characteristics with genomic aberrations were tested with χ^2 tests or Cochran Armitage tests for trend. P values were considered significant for values <0.05. The Benjamini-Hochberg approach was used to adjust for multiple comparisons.

Supplementary Figures

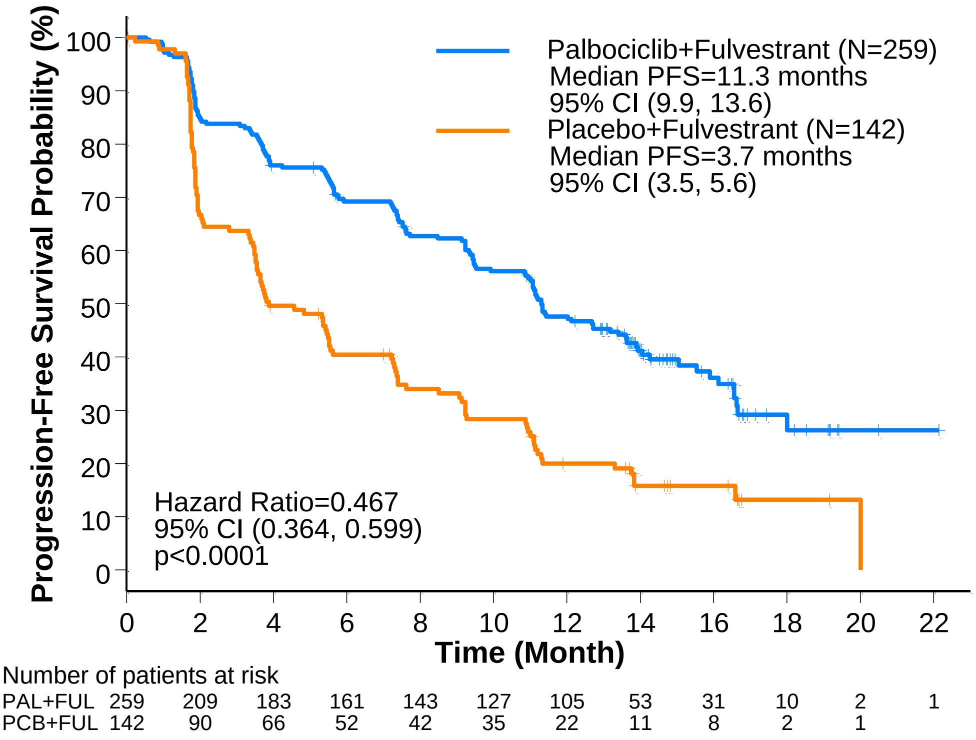

Supplementary figure 1. Progression free survival in the subset of patients assessed for copy number and circulating tumor fraction (n = 401).

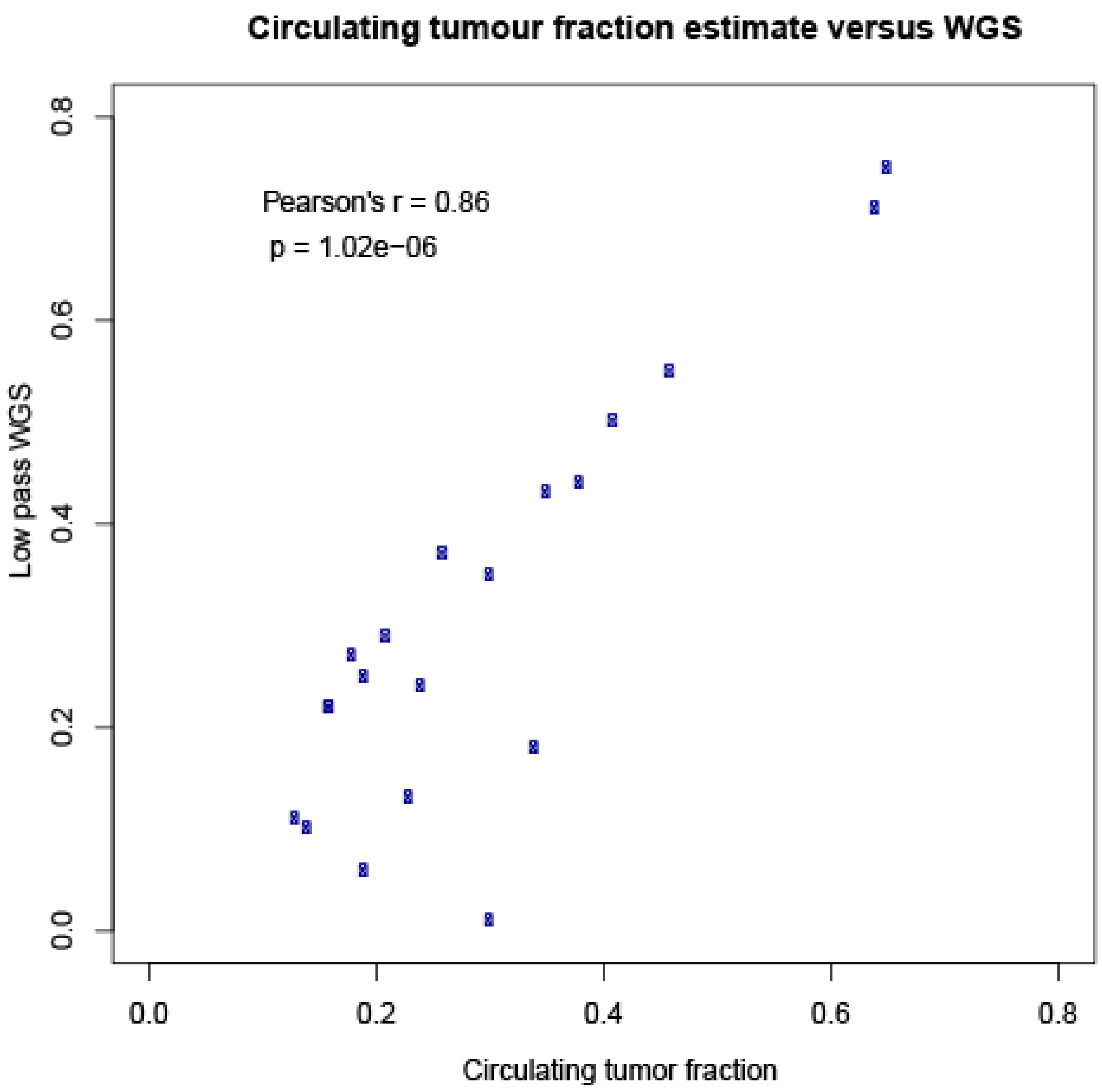

Supplementary figure 2. Comparison of circulating tumor fraction/purity estimates from the purity panel and from low pass whole genome sequencing using ichorCNA.

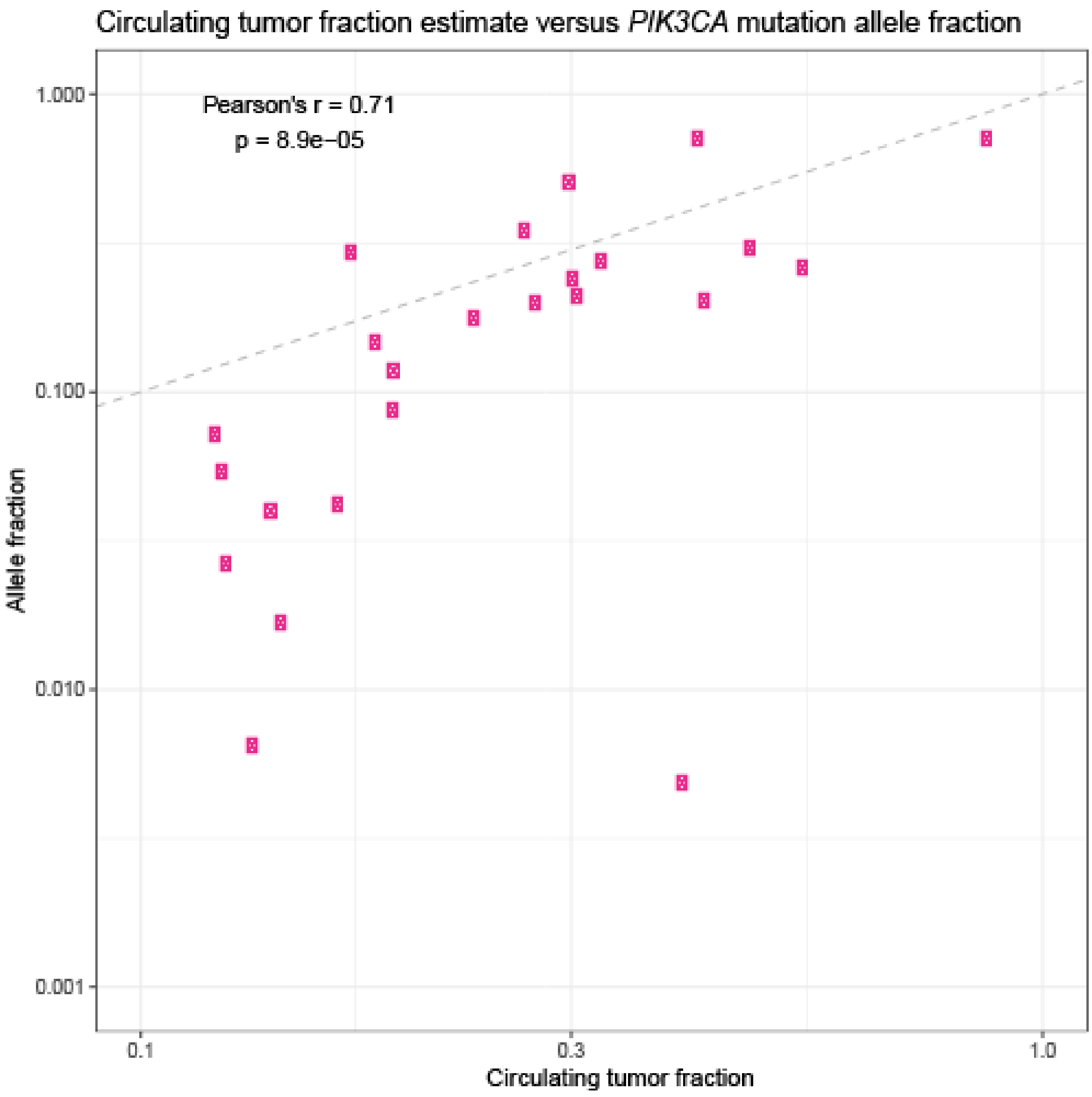

Supplementary figure 3. Comparison of allele fraction of *PIK3CA* mutations with circulating tumor fraction estimates from ctDNA sequencing. *PIK3CA* allele fractions rarely exceed tumor fraction estimates, with high correlation in the majority of mutations reflecting truncal mutations. A minority of cancers with *PIK3CA* mutations have lower allele fractions, in part reflecting likely sub-clonal mutations, and in part at low circulating tumor fractions reflecting stochastic effects.

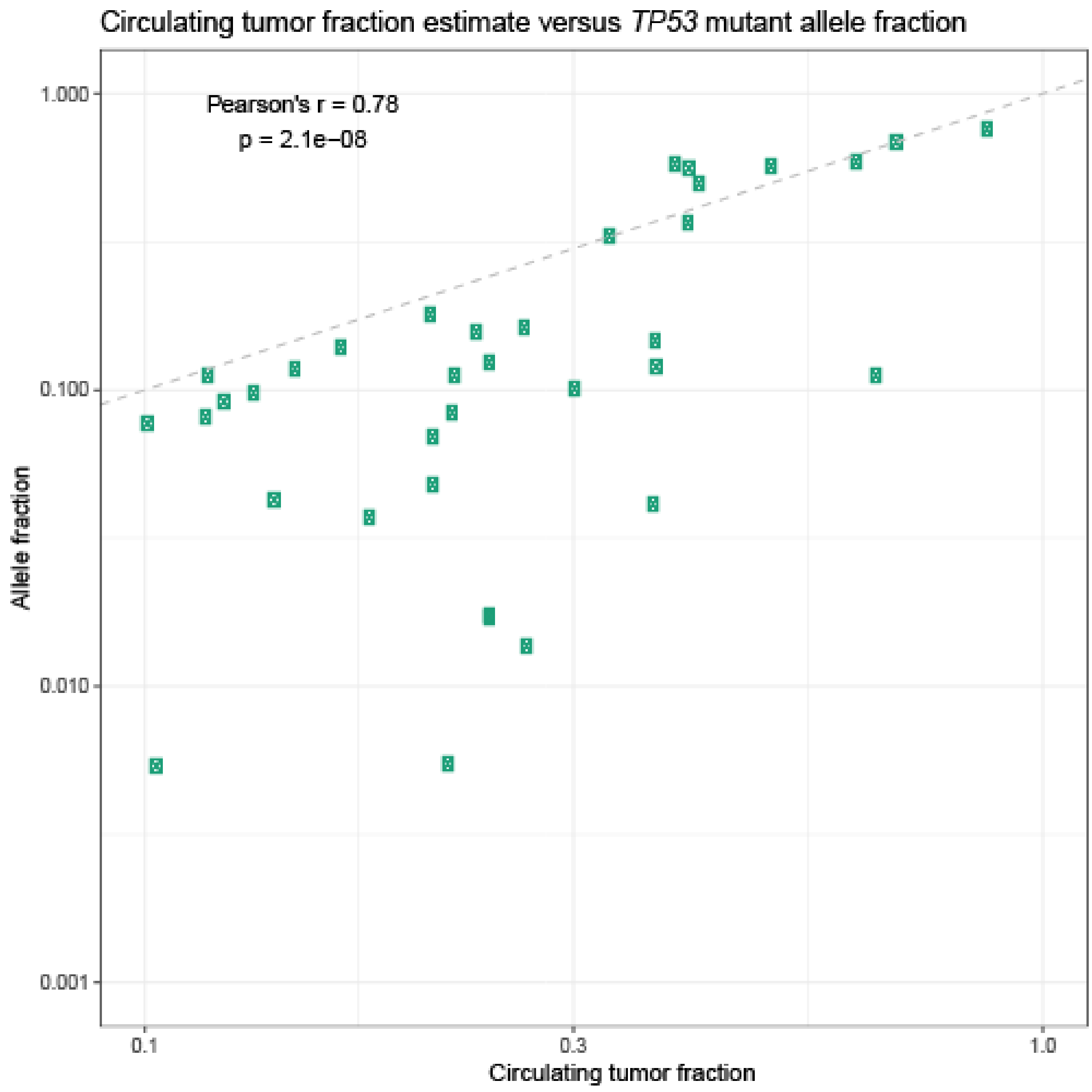

Supplementary figure 4. Comparison of allele fraction of *TP53* mutations with circulating tumor fraction estimates from sequencing. *TP53* allele fractions rarely exceed tumor fraction estimates, with high correlation in the majority of mutations reflecting truncal mutations. A minority of cancers with *TP53* mutations have lower allele fractions, in part reflecting likely sub-clonal mutations, and in part at low circulating tumor fractions reflecting stochastic effects.

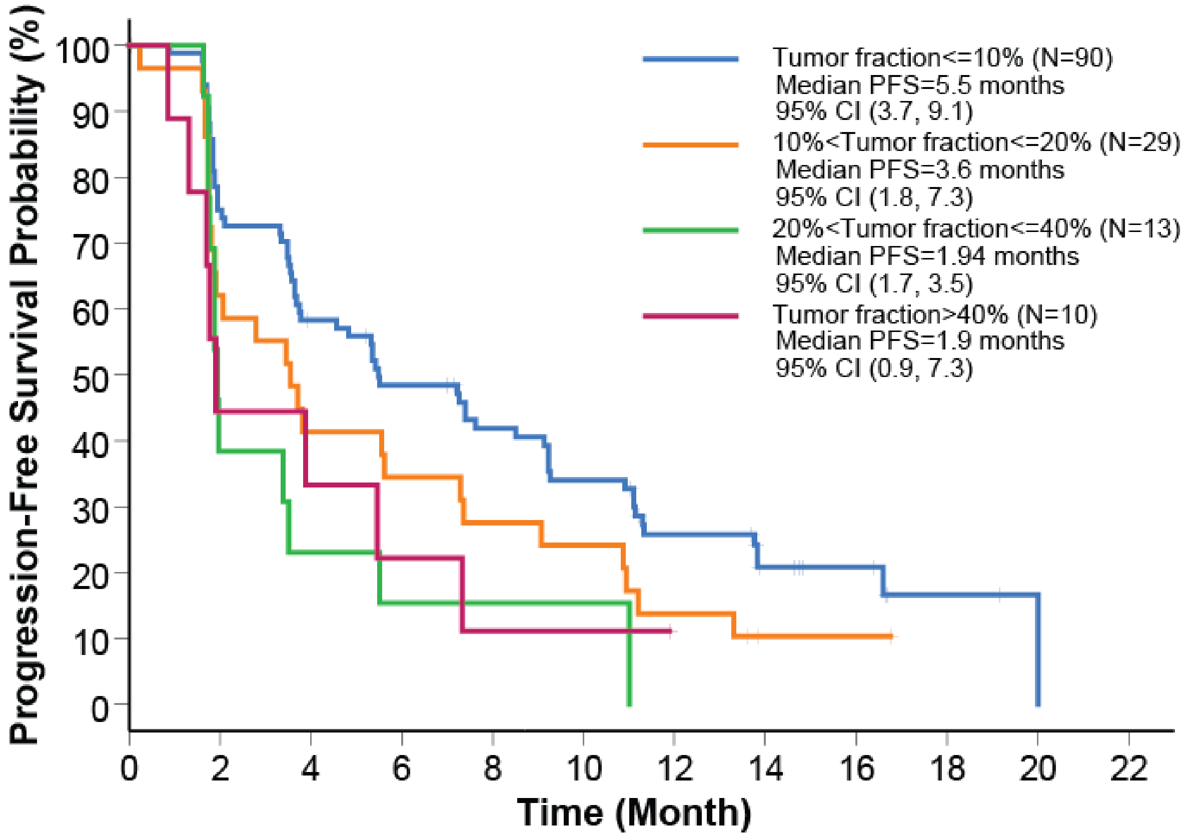

Supplementary figure 5. Progression free survival for whole cohort assessed for circulating tumor fraction separated by 10%, 20% and 40%. P value is log rank.

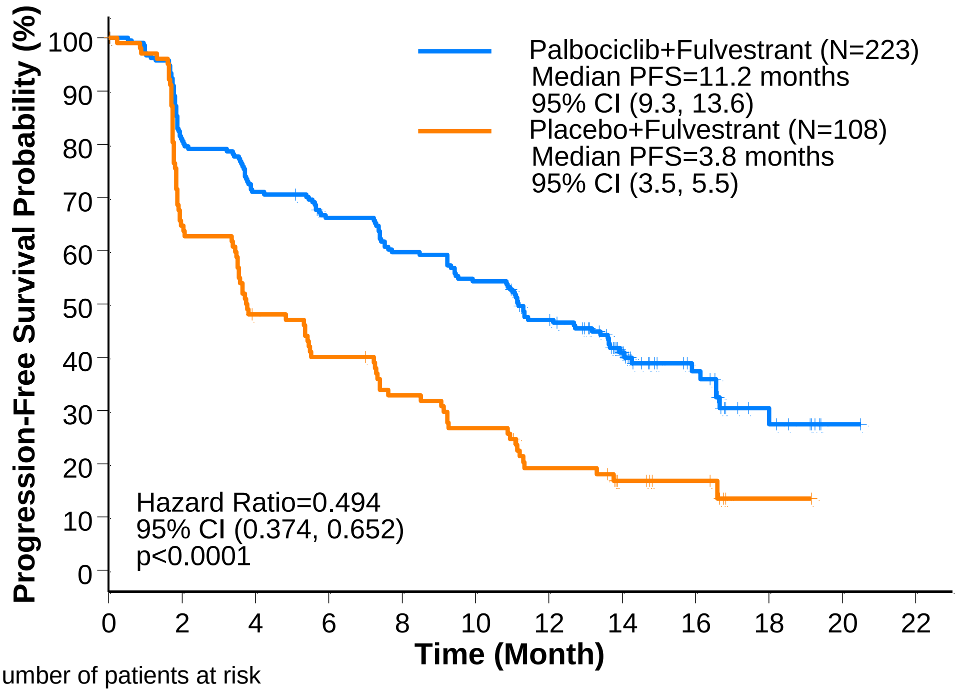

Number of patients at risk

PAL+FUL	223	171	148	134	120	108	90	44	25	10	1
PCB+FUL	108	66	48	40	32	26	17	10	7	1	

Supplementary figure 6. Progression free survival in the subset of patients assessed for mutations (n = 331). P value is logrank. CI - confidence interval, PFS - progression free survival, PAL – palbociclib, PCB – placebo, FUL – fulvestrant.

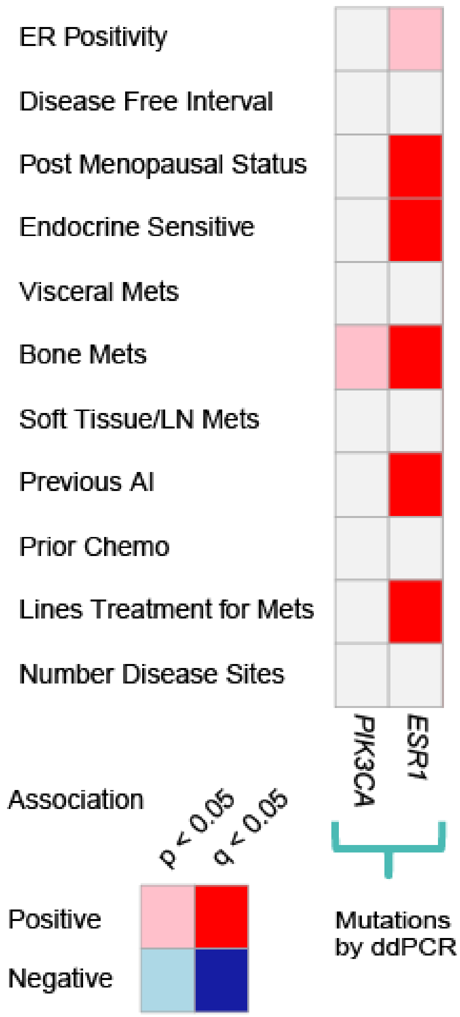

Supplementary figure 7. Associations between clinical characteristics and *PIK3CA* and *ESR1* status by digital PCR. Significance assessed with Chi-squared for categorical or Cochran-Armitage where a category was ordinal.

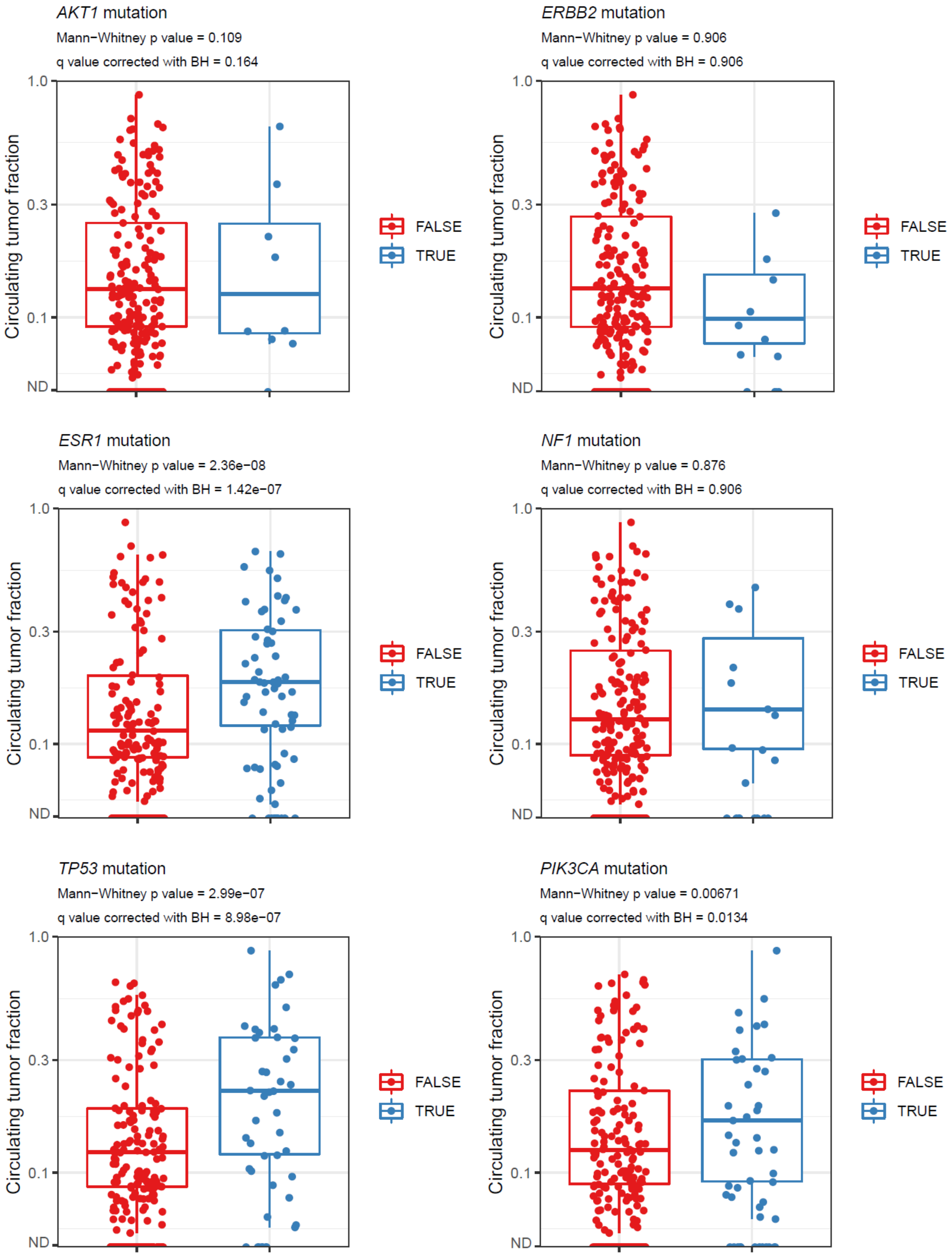

Supplementary figure 8. Association between circulating tumor fraction and individual mutations. Only alterations found with >5 incidences at baseline included, comparing fraction in patients with mutation detected in the indicated gene (TRUE) compared to all the other patients without the mutation detected (FALSE).

The cohort is the n = 310 patients with data for both mutations and circulating tumor fraction. P values calculated using Mann-Whitney test. ND – not detected. BH – Benjamini-Hochberg correction.

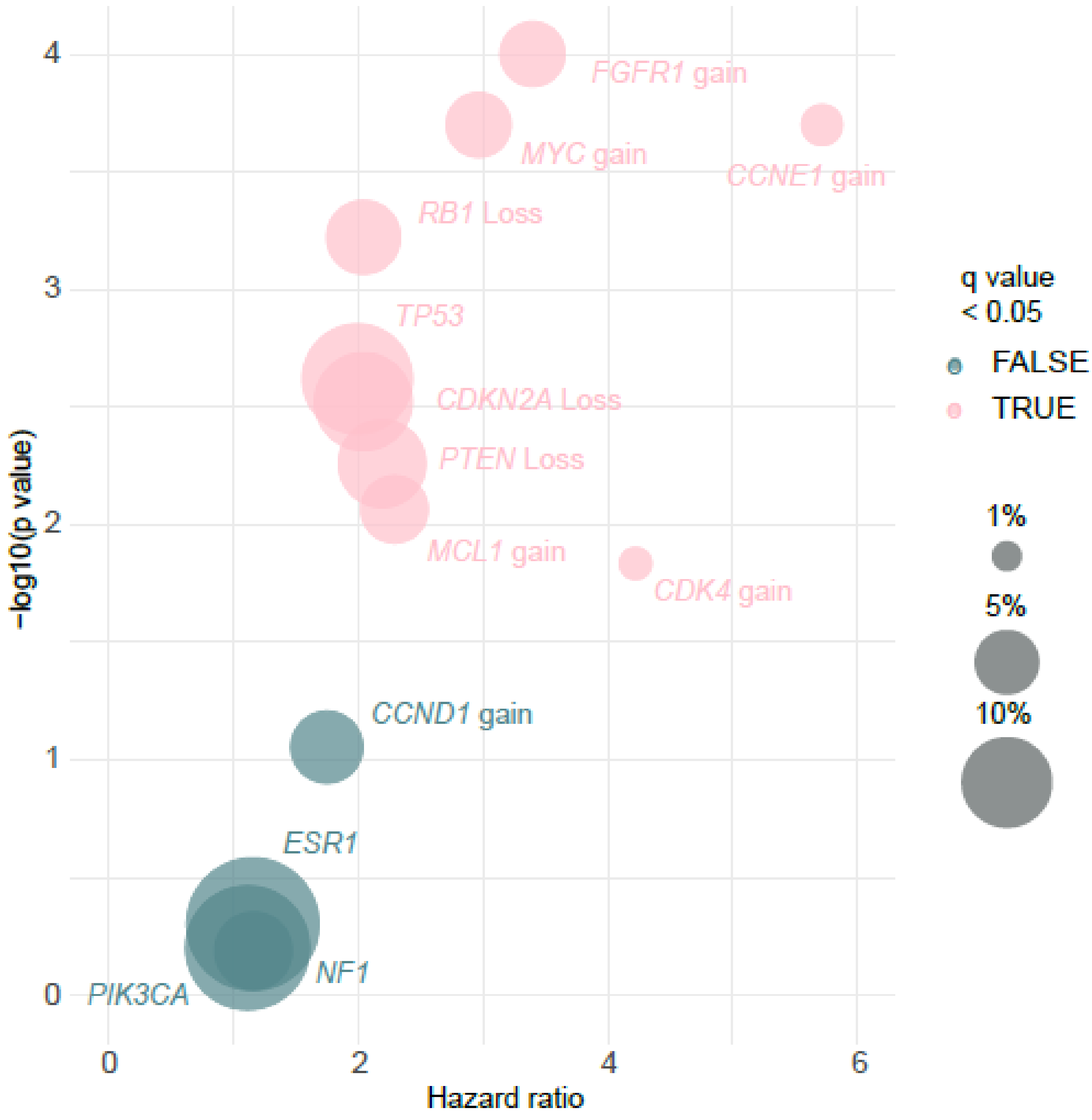

Supplementary figure 9. Univariable survival analysis for mutations and copy number aberrations, gains and losses/LOH, in the palbociclib plus fulvestrant arm (n = 223 for mutations 259 for copy number). P values are log rank and corrected with the Benjamini-Hochberg method.

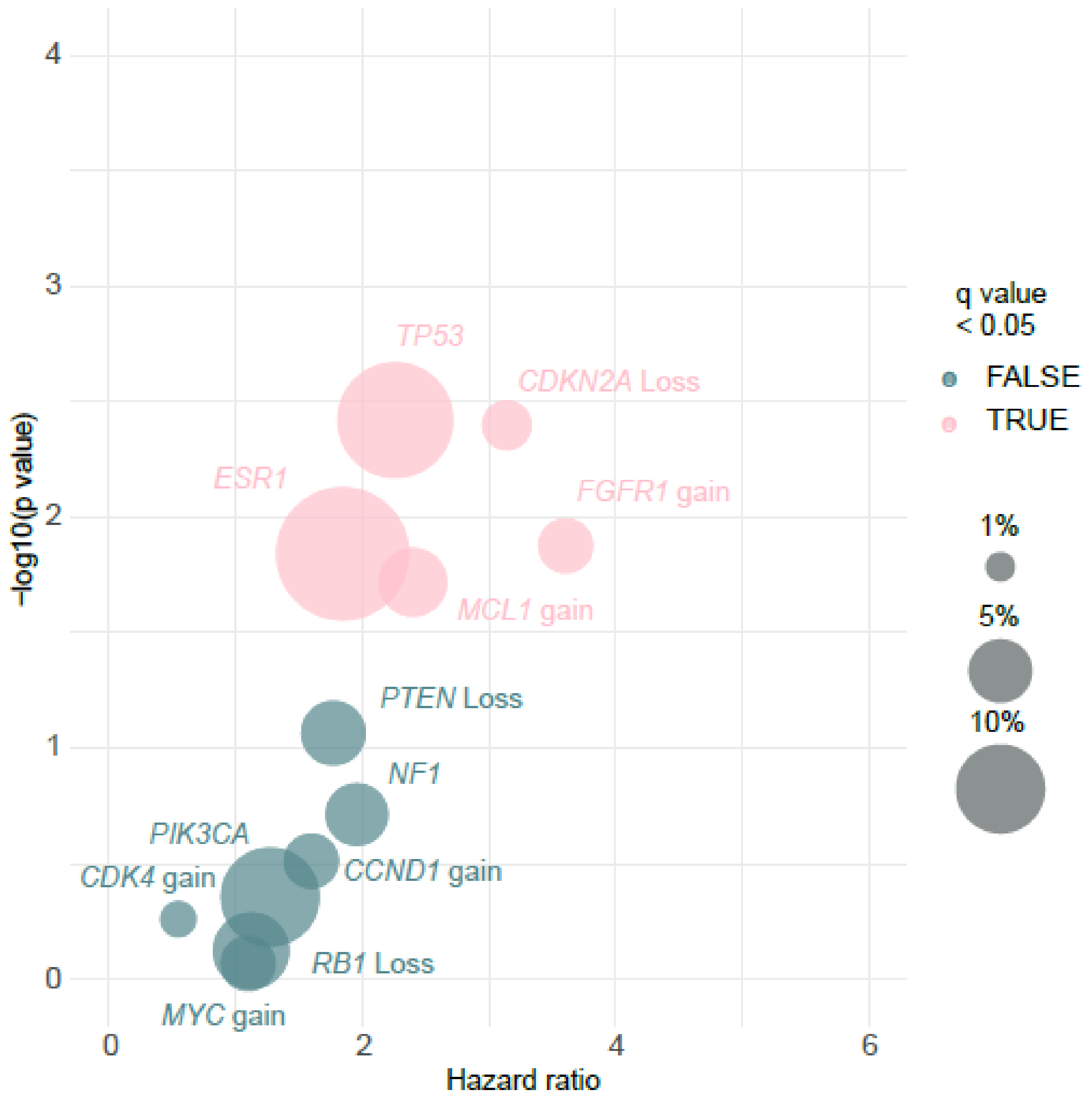

Supplementary figure 10. Univariable survival analysis for mutations and copy number aberrations, gains and losses/LOH, in the placebo plus fulvestrant arm (n = 108 for mutations 142 for copy number). P values are log rank and corrected with the Benjamini-Hochberg method.
